# Effects of *Piper aduncum* (Piperales: Piperaceae) Essential Oil and Its Main Component Dillapiole on Detoxifying Enzymes and Acetylcholinesterase Activity of *Amblyomma sculptum* (Acari: Ixodidae)

**DOI:** 10.3390/ijms25105420

**Published:** 2024-05-16

**Authors:** Adalberto Alves Pereira Filho, Vladimir Fazito do Vale, Caio Marcio de Oliveira Monteiro, Mayara Macedo Barrozo, Mariana Alves Stanton, Lydia Fumiko Yamaguchi, Massuo Jorge Kato, Ricardo Nascimento Araújo

**Affiliations:** 1 Laboratório de Artrópodes Hematófagos, Departamento de Parasitologia/ICB, Universidade Federal de Minas Gerais, Belo Horizonte 31270-901, MG, Brazil; 2 Grupo de Pesquisa Triatomíneos, Instituto René Rachou, Fiocruz, Belo Horizonte 30190-009, MG, Brazil; vladimir.vale@fiocruz.br; 3 Centro de Parasitologia Veterinária, Escola de Veterinária e Zootecnia, Instituto de Patologia Tropical e Saúde Pública, Universidade Federal de Goiás, Goiânia 74690-900, GO, Brazil; caiosat@gmail.com (C.M.d.O.M.); mayarabarrozo@discente.ufg.br (M.M.B.); 4 Laboratório de Química de Produtos Naturais, Departamento de Química Fundamental, Instituto de Química, Universidade de São Paulo, São Paulo 05424-140, SP, Brazil; mariana.a.stanton@gmail.com (M.A.S.); lydyama@gmail.com (L.F.Y.); massuojorge@gmail.com (M.J.K.)

**Keywords:** *Amblyomma cajennense* complex, α-esterase, β-esterase, glutathione-S-transferase, natural products

## Abstract

*Amblyomma sculptum* is a species of tick in the family Ixodidae, with equids and capybaras among its preferred hosts. In this study, the acaricidal activity of the essential oil (EO) from *Piper aduncum* and its main component, Dillapiole, were evaluated against larvae of *A. sculptum* to establish lethal concentration values and assess the effects of these compounds on tick enzymes. Dillapiole exhibited slightly greater activity (LC_50_ = 3.38 mg/mL; 95% CI = 3.24 to 3.54) than *P. aduncum* EO (LC_50_ = 3.49 mg/mL; 95% CI = 3.36 to 3.62) against ticks. The activities of α-esterase (α-EST), β-esterase (β-EST), and glutathione-S-transferase (GST) enzymes in *A. sculptum* larvae treated with Dillapiole showed a significant increase compared to the control at all concentrations (LC_5_, LC_25_, LC_50_ and LC_75_), similar results were obtained with *P. aduncum* EO, except for α-EST, which did not differ from the control at the highest concentration (LC_75_). The results of the acetylcholinesterase (AChE) activity show an increase in enzyme activity at the two lower concentrations (LC_5_ and LC_25_) and a reduction in activity at the two higher, lethal concentrations (LC_50_ and LC_75_) compared to the control. These results suggest potential mechanisms of action for these natural acaricides and can provide guidance for the future development of potential plant-derived formulations.

## 1. Introduction

*Amblyomma sculptum* Berlese, 1888 is a species of tick commonly found in the most populated states of Brazil, as well as Paraguay and northern Argentina [1]. Its preferred hosts are equids, but it can also parasitize cattle, other domestic animals, and wildlife [2,3]. The species is part of a specific complex called *Amblyomma cajennense* sensu lato, with a wide distribution in the Americas [4].

Of great importance is its ability to transmit pathogens during the blood-feeding process on vertebrate hosts. In the scientific field, it is well-established that *A. sculptum* serves as a vector for the bacterium *Rickettsia rickettsii* Lima, 1916, which can be transmitted to humans when an infected tick feeds on human blood, leading to Brazilian Spotted Fever (BSF) [5,6,7,8]. Recent years have seen reported cases of BSF in Brazil, highlighting the urgent need for alternative tick control methods [9,10]. 

In the search for alternatives for acaricidal activity on *A. sculptum*, essential oils (EOs) and isolated compounds from EOs can represent excellent sources of biologically active natural products [11,12]. Notably, their toxicity is considerably lower, making them less harmful to the environment when compared to synthetic acaricides available on the market [13]. Additionally, they exhibit more environmentally friendly behavior, i.e., they are biodegradable and potentially reduce ecological impact compared to synthetic alternatives [14,15,16].

*Piper aduncum* L. is an example of a plant whose EO has potential acaricidal activity [17,18,19], which makes this species a promising candidate for tests against *A. sculptum*. This plant is a shrubby plant belonging to the Piperaceae family that grows 6 to 7 m tall with lance-shaped leaves, native to southern Mexico, the Caribbean, and abundant in South and Central America [20,21]. 

The ability to detoxify compounds with insecticidal/acaricidal activities is associated with an increase in the expression or activity of specific enzyme families, such as esterases (EST) and glutathione-S-transferases (GST), and these are the possible resistance mechanisms in arthropods [22,23,24,25]. Consequently, monitoring the levels of these enzymes is of utmost importance for evaluating the potential use of new substances derived from plants with acaricidal activity.

Another important enzyme to be evaluated in the proposal of new acaricidal substances is acetylcholinesterase (AChE), whose action is crucial in the propagation of nerve impulses. Inhibition of AChE is reported as one of the main activities of organophosphates and carbamates [26,27]. However, mutations can produce ticks that are resistant to this class of insecticides, altering the enzyme’s structure in such a way that it is no longer effectively inhibited by the insecticides [28]. There are data in the literature demonstrating that natural compounds found in EOs, such as thymol and carvacrol, can increase the activity of enzymes related to the detoxification process in the cattle tick, *Rhipicephalus microplus* Canestrini, 1887 [29]. As part of an effort to contribute to the sustainable, economical, and ecological reduction in tick populations, the study of plants with acaricidal activity has been gaining prominence in research. Therefore, the present study aims mainly to evaluate the acaricidal activity of the EO of *P. aduncum* and its main compound, Dillapiole, on larvae of *A. sculptum*, establish lethal concentration values, and assess the effects of these compounds on some tick enzymes.

## 2. Results

### 2.1. Oil Composition Analysis

**Figure 1:**
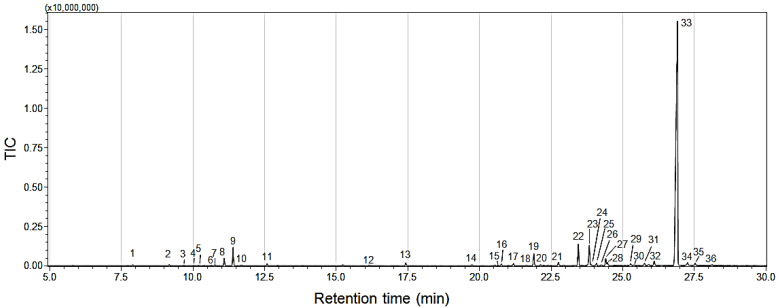
The GC-MS chromatograms of the essential oil (EO) of *Piper aduncum* show that Compound 33 corresponds to the major compound Dillapiole. The remaining compounds are identified in Table 1.

The EO of *P. aduncum* was analyzed by GC-MS to determine its main constituents. Identification of these constituents was based on library search, retention index (RI), and, when available, the use of standard compounds. The relative percentages of each constituent are presented in Table 1. A total of thirty-six compounds were identified in the oil, which were classified as hydrocarbon monoterpenes, hydrocarbon sesquiterpenes, oxygenated monoterpenes, oxygenated sesquiterpenes, and phenylpropanoids. The major compound in the EO, determined through GC-MS analysis, was the phenylpropanoid Dillapiole (81.9% relative concentration), which was selected for use in the tests (Figure 1).

### 2.2. Effect of Piper aduncum EO and Dillapiole on Larval Mortality

The effect of *P. aduncum* EO and its major constituent, Dillapiole, against *A. sculptum* larvae was evaluated after 24 h of treatment, following the conditions described in Section 4.3. Dillapiole exhibited greater activity (LC_50_ = 3.38 mg/mL; 95% CI = 3.24 to 3.54) compared to *P. aduncum* EO (LC_50_ = 3.49 mg/mL; 95% CI = 3.36 to 3.62); however, there was no significant difference between the EO and the main compound due to overlapping confidence intervals. The lethal concentrations (LC_5_, LC_25_, LC_50_, and LC_75_) of the compounds for the larvae are listed in Table 2.

### 2.3. Effect of Piper aduncum EO and Dillapiole on Detoxifying Enzymes of Larvae

**Figure 2:**
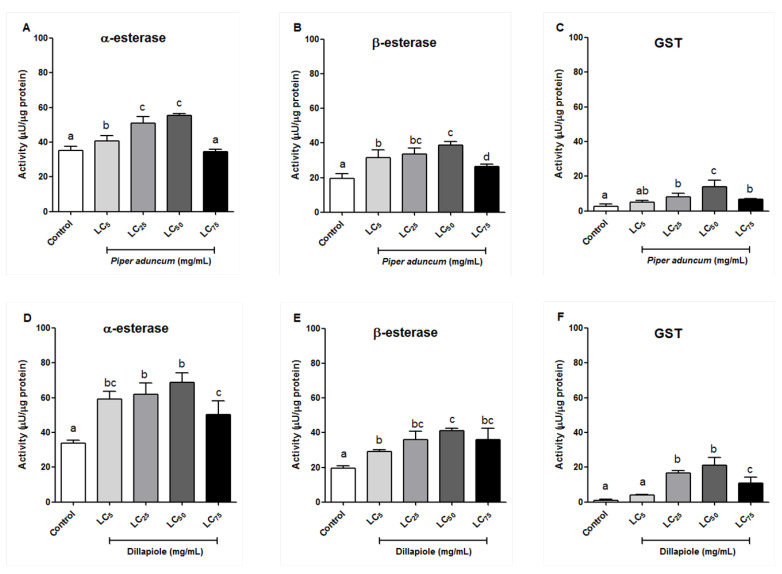
Effect of the essential oil (EO) of *Piper aduncum* (**A**–**C**) and of Dillapiole (**D**–**F**) on the enzymatic activity of α-esterase (α-EST), β-esterase (β-EST), and glutathione-S-transferase (GST) of *Amblyomma sculptum* larvae. Means with the different letters are significantly different from each other (*p* < 0.05) in an ANOVA followed by Tukey post hoc test. Bars represent the mean ± standard deviation of 5 biological replicates.

The effects of *P. aduncum* EO and of its major constituent, Dillapiole, on the detoxifying enzymes of *A. sculptum* larvae were evaluated under the conditions described in Section 4.4.2 and Section 4.4.3. The activities of α-esterase (α-EST), β-esterase (β-EST), and glutathione-S-transferase (GST) enzymes in the *A. sculptum* larvae treated with *P. aduncum* EO and Dillapiole showed a significant increase compared to the control group at all concentrations of *P. aduncum* EO and Dillapiole tested, with the exception of α-EST treated with the LC_75_ of *P. aduncum* EO (Figure 2), which did not significantly differ from the control. 

At the highest concentration (LC_75_: 5.05 mg/mL) of *P. aduncum* EO, a relative decrease in the activity of the three enzymes was observed when compared to lower concentrations (α-EST: 34.38 ± 1.62 µU/µg; β-EST: 26.35 ± 1.28 µU/µg; and GST: 6.86 ± 0.18 µU/µg), suggesting the higher concentration may inhibit enzyme activity, or not cause an increase, in the case of α-EST after *P. aduncum* EO treatment. A similar effect was observed at the highest concentration (LC_75_: 4.69 mg/mL) of Dillapiole (α-EST: 50.47 ± 7.58 µU/µg; β-EST: 36.10 ± 6.51 µU/µg; and GST: 10.81 ± 3.41 µU/µg), where the increase in enzyme activity (relative to the control) was lower than in other concentrations (Figure 2).

### 2.4. Effect of Piper aduncum EO and Dillapiole on Acetylcholinesterase (AChE) Activity of Larvae

**Figure 3:**
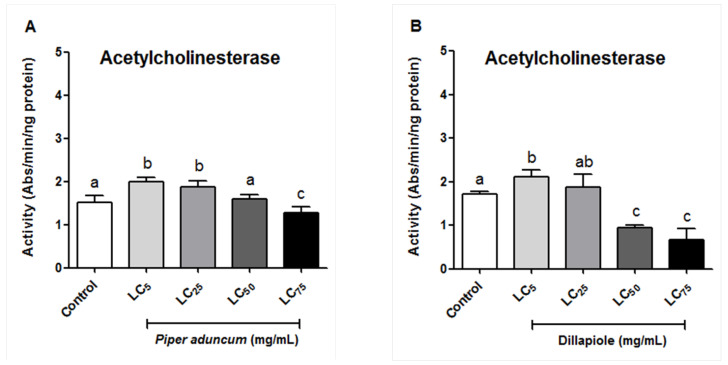
Effect of the essential oil (EO) of *Piper aduncum* (**A**) and of Dillapiole (**B**) on acetylcholinesterase activity of *Amblyomma sculptum* larvae. Means with the different letters are significantly different from each other (*p* < 0.05) in an ANOVA followed by Tukey’s post hoc test. Bars represent the mean ± standard deviation of 5 biological replicates.

The effects of *P. aduncum* EO and its major constituent, Dillapiole, on the AChE activity of *A. sculptum* larvae were evaluated under the conditions described in Section 4.4.4. In our experiments, both *P. aduncum* EO and Dillapiole treatment caused an initial increase in enzyme activity at lower concentrations (LC_5_ and LC_25_) in contrast with a reduction in AChE activity at higher concentrations (LC_50_ and LC_75_), compared to controls, with the exception of the AChE activity of the larvae treated with *P. aduncum* EO at LC_50_ (3.49 mg/mL; AChE: 1.6 ± 0.08 Abs/min/ng protein) which did not differ significantly from the control group (AChE: 1.5 ± 0.15 Abs/min/ng protein) (Figure 3). 

### 2.5. Scanning Electron Microscopy

**Figure 4:**
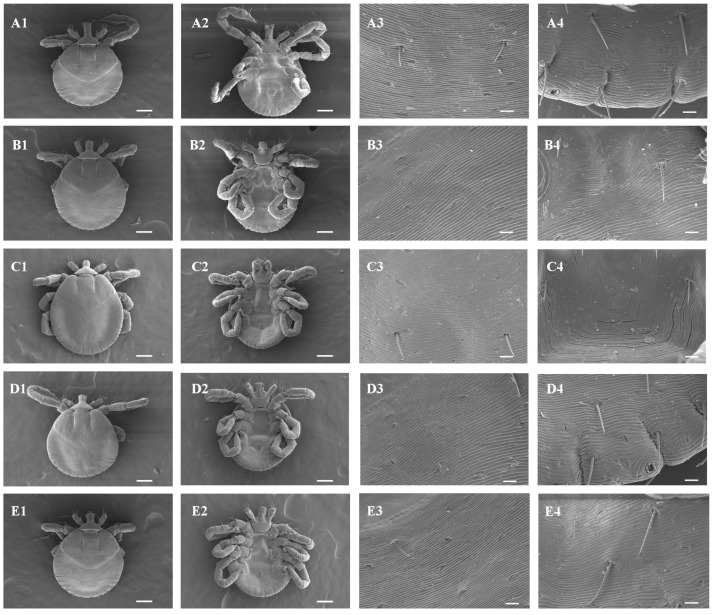
Scanning electron microscopy images of the cuticle of *Amblyomma sculptum* larvae after incubation with different treatments. Vehicle Control (1% acetone and 0.02%Triton X-100)—(**A1**) (Dorsal view × 120), (**A2**) (Ventral view × 120), (**A3**) (Dorsal view × 1000), and (**A4**) (Ventral view × 1000); *Piper aduncum* (LC 50 mg/mL)—(**B1**) (Dorsal view × 120), (**B2**) (Ventral view × 120), (**B3**) (Dorsal view × 1000), and (**B4**) (Ventral view × 1000); *Piper aduncum* (LC 75 mg/mL)—(**C1**) (Dorsal view × 120), (**C2**) (Ventral view × 120), (**C3**) (Dorsal view × 1000), and (**C4**) (Ventral view × 1000); Dillapiole (LC 50 mg/mL)—(**D1**) (Dorsal view × 120), (**D2**) (Ventral view × 120), (**D3**) (Dorsal view × 1000), and (**D4**) (Ventral view × 1000); or Dillapiole (LC 75 mg/mL)—(**E1**) (Dorsal view × 120), (**E2**) (Ventral view × 120), (**E3**) (Dorsal view × 1000), and (**E4**) (Ventral view × 1000). (**A1**,**A2**,**B1**,**B2**,**C1**,**C2**,**D1**,**D2**,**E1**,**E2**): scale bar = 100 µm and (**A3**,**A4**,**B3**,**B4**,**C3**,**C4**,**D3**,**D4**,**E3**,**E4**): scale bar = 10 µm.

Ten images were obtained from the larvae in each treatment group, with five ventral and five dorsal views under the conditions described in Section 4.5. Only one image from each group (dorsal and ventral) was selected for data presentation (Figure 4). The scanning electron microscopy did not reveal cuticle alterations in *A. sculptum* larvae after in vitro exposure to *P. aduncum* EO and Dilapiolle (Figure 4). The cuticles remained intact, like what was observed in the vehicle control group.

## 3. Discussion

While there are few confirmed records of resistance to the acaricides used for the controlling of the *Amblyomma* complex until now [30,31,32], it is important to note that resistance to insecticides and acaricides is a phenomenon that can develop over time due to the selective pressure exerted by these chemicals [26,33]. Thus, the search for acaricides derived from EOs and isolated compounds has led to an increase in the number of studies that have caught the attention of the scientific community [13]. The present study showed that the EO of *P. aduncum* and its main component have an acaricidal effect on *A. sculptum* and interfere with the activity of detoxifying enzymes and AChE. These results reinforce what has been shown in the literature regarding the acaricidal activity of this *Piper* species [17]. This also is the first time the effect of *P. aduncum* EO and of the phenylpropanoid Dillapiole have been shown on important detoxifying enzymes (GST, α-Est, β-Est) and on AChE in the tick *A. sculptum*.

The EO of *P aduncum* contains more than 30 compounds, most of which are terpenes that are present in very low concentrations and together represent less than 20% of the essential oil content. The major component in the *P. aduncum* EO, the phenylpropanoid Dillapiole (81.9% of EO content), was selected for use in our tests. Dillapiole on its own exhibited greater activity (LC_50_ = 3.38 mg/mL; 95% CI = 3.24 to 3.54) than *P. aduncum* EO (LC_50_ = 3.49 mg/mL; 95% CI = 3.36 to 3.62); however, there was no significant difference between the EO and the major component. The acaricidal potential of *P. aduncum* EO has already been demonstrated in other tick species such as *R. microplus*, where it resulted in 100% mortality in larvae at a concentration of 0.1 mg/mL [17]. Regarding Dillapiole, to our knowledge, there is currently no data on acaricidal activity in any species. However, it has been reported that phenylpropanoids such as (*E*)-cinnamaldehyde [34] and Eugenol [12] have acaricidal activity on larvae of *A. sculptum*. This may indicate the possible susceptibility of *A. sculptum* larvae to compounds of the phenylpropanoid class. However, testing with other compounds of this class on *A. sculptum* larvae is necessary to corroborate this hypothesis.

The need to perform biological activity tests with the major compound(s) found in the chromatographic analysis of an EO is necessary due to two main reasons: the first is that EOs can exhibit variations in their compound composition due to varying environmental conditions, soil composition, growth stages, biotic factors, and genetic diversity within the plant [35,36,37,38]. The second reason is that EOs are complex mixtures of monoterpenes, sesquiterpenes, and phenylpropanoids, and the primary components can interact synergistically or antagonistically with minor constituents, thereby producing results that differ from what might be expected for individual compounds [39]. Another advantage of compounds found EOs is their standardization and the ease of obtaining the active ingredient on a large scale for developing commercial formulations [13]. Based on the data obtained from the Acaricidal Effect X Chromatographic Analysis, we conclude that there seems to be no significant interaction (antagonistic or synergistic) of the major molecule, Dillapiole, with the other minor components present in the *P. aduncum* EO.

An increase in the activity of detoxifying enzymes (GST, α-EST, β-EST) was observed for *A. sculptum* larvae treated with *P. aduncum* EO and pure Dillapiole, reaching its peak at the sublethal concentration (LC_50_). This elevation in the activity of detoxifying enzymes from arthropods is, in general, a result of their defense mechanisms against certain substances [40,41]. Similar to the results obtained in this study, a significant increase in GST activity was found in a susceptible strain of *R. microplus* (Porto Alegre) when treated with sublethal concentrations (LC_1_, LC_25_, and LC_50_) of two monoterpenes (carvacrol and thymol) [29]. In arthropods, GSTs are a set of versatile enzymes engaged in oxidative protection that confer resistance via direct metabolism or sequestration of chemicals, but also indirectly by providing protection against oxidative stress induced by insecticide exposure [42,43]. A notable reduction in the activity of the analyzed detoxifying enzymes, GST, α-EST, and β-EST, was evident in the *A. sculptum* larvae after exposure to the LC_75_ of both *P. aduncum* EO and Dillapiole, when compared to the larvae treated with the LC_50_, and in one case (α-EST activity after treatment with LC_75_ of both *P. aduncum* EO and Dillapiole) showing a lack of increase in enzyme activity compared to the control. This reduction in enzyme activity after treatment can be attributed to the higher exposure concentration, which likely facilitates the entrance of a greater quantity of toxic substances into the tissues, causing increased oxidative stress and, consequently, reducing the levels of antioxidant enzyme activity [29].

In our experiments, both *P. aduncum* EO and Dillapiole progressively inhibited the activity of AChE in the larvae of *A. sculptum* when applied at higher concentrations, with increased inhibition upon exposure to the LC_75_. This suggests that in addition to affecting the activity of detoxification enzymes, both *P. aduncum* EO and Dillapiole may also interfere with the tick’s neurotransmission. In the literature, EOs from the leaves and stems of *Piper austrosinense*, *Piper puberulum*, *Piper flaviflorum*, *Piper betle*, and *Piper hispidimervium* have shown robust AChE inhibitory activity in vitro, with IC_50_ values ranging from 1.51 to 13.9 mg/mL [44]. In other arthropods such as *Aedes aegypti*, *A. albopictus*, and *Culex quinquefasciatus*, phenylpropanoids Asaricin 1 and Isoasarone 2 displayed potent inhibition on acetylcholinesterase, with relative IC_50_ values of 0.73 to 1.87 μg/mL, respectively [45]. 

**Figure 5:**
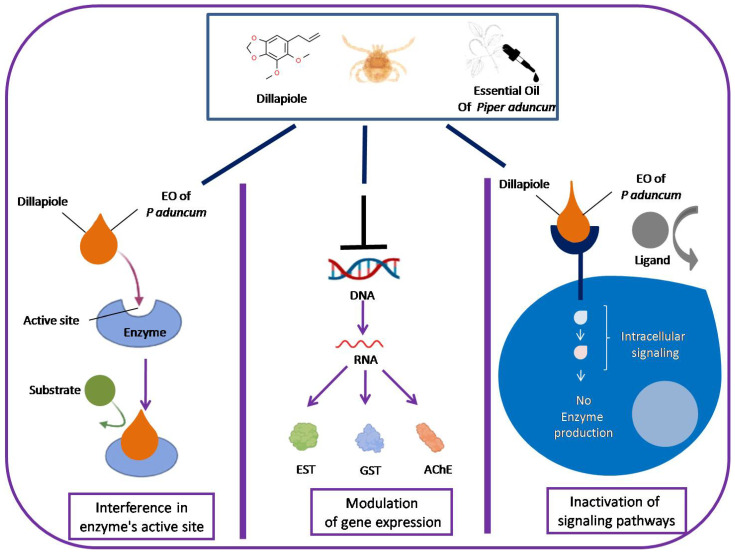
Overview of three hypotheses that can explain the potential mechanisms by which *P. aduncum* EO and Dillapiole disrupt certain enzymes in the present study. The chemical structure of dillapiole was obtained from the ChemSpider website. Some figure templates used in this overview were obtained from the BioRender software©2024.

Three hypotheses can explain the potential mechanisms by which *P. aduncum* EO and Dillapiole disrupt certain enzymes in the present study: 1—*P. aduncum* EO and its major component may inhibit intracellular signaling pathways that regulate the production of detoxifying enzymes, for example, modulating intracellular signaling cascades; 2—*P. aduncum* EO and Dillapiole may influence the expression of genes that encode detoxifying enzymes; 3—*P. aduncum* EO and Dillapiole may directly bind to the active site of the enzymes under study, thereby blocking their enzymatic activity. In this way, future studies are necessary to test these hypotheses and to bring clarity to the ideas regarding the relationship between new compounds and specific enzyme families (Figure 5).

At the enzymatic level, after data analysis, the EO of *P. aduncum* and Dilapiolle have the potential to alter the enzymatic activity of important detoxifying enzymes and of neurotransmission in *A. sculptum* larvae. At the morphological level, the investigation sought to determine whether the EO of *P. aduncum* and its major compound could affect the cuticle of *A. sculptum*, using scanning electron microscopy. In the current literature, it is already known that *P. aduncum* EO has the ability to destroy the cuticle of arthropods, as evidenced by the fact that in *A. aegypti*, the EO of this species was able to cause a strong rupture throughout the extent of the cuticle [46]. Based on this idea, we sought to verify whether *P. aduncum* EO and its major compound could affect the cuticle of *A. sculptum*. No morphological alterations at the cuticle level were observed in both groups (treated vs. control). The EO of *P. aduncum* and its major compound did not cause changes at the cuticle level, although damage may have occurred in the internal tissues and organs, as there was a reduction in the activity of antioxidant enzymes, as discussed above. Therefore, future studies focusing on the internal tissues of the tick are necessary for a better understanding and verification of these compounds in the action on tick tissues.

## 4. Materials and Methods

### 4.1. Plant Material, Identification, and Extraction of Essential Oils

The leaves of *Piper aduncum* were collected in the period of January to June 2020 in the Institute of Chemistry—USP (46°43′32″ W; 23°33′54″ S). A specimen of the plant was deposited at the Herbário do Jardim Botânico do Rio de Janeiro under registration (K-0057), and the identification was made by Dr. Elsie Franklin Magalhães. The specimen collection was made under protocol number #59161-1 from SISBIO (Sistema de Autorização e Informação em Biodiversidade).

The EO was extracted from fresh leaves and submitted to hydrodistillation in a Clevenger-type apparatus for 4 h, using 300–500 g of fresh leaves and 500 mL of distilled water [47,48]. The EO was collected and dried with anhydrous sodium sulfate and stored in amber bottles in a refrigerator at 4 °C until the experiments and analysis by GC-MS were performed according to the method described elsewhere [46].

The main compound from *P. aduncum* EO characterized by GC-MS and used in this study was Dillapiole. Pure Dillapiole was obtained by fractionation using an Isolera Flash Chromatography system (Biotage INC) according to the method described in [46].

### 4.2. Tick Collection

Engorged *A. sculptum* females were obtained from naturally infested horses at the Veterinary School of UFMG farm, located in the municipality of Pedro Leopoldo, MG, Brazil. They were maintained in a biochemical oxygen demand (BOD) incubator under a controlled temperature of 28 ± 2 °C and 90 ± 5% humidity until oviposition. Eggs laid in the lab were collected and placed in 15 mL conical-bottom tubes closed with cotton to allow air and moisture flow. Subsequently, they were incubated under the same temperature and humidity conditions described above. This population has been maintained in the laboratory for over 10 years until the present day and has been characterized as susceptible to synthetic acaricides of the pyrethroid class according to the FAO guidelines [49]. The colony is maintained at the Laboratory of Hematophagous Arthropods—LAH, following the recommendations of the Ethics Committee (CEUA-UFMG) at the Federal University of Minas Gerais, Brazil, under protocol number 60/2020.

### 4.3. Larval Immersion Test

The larval immersion test (LIT) was performed following the method described by Sabatini et al., 2001 [50]. *Piper aduncum* EO and Dillapiole were tested at concentrations of 0.6; 0.84; 1.2; 1.7; 2.4; 3.5; 5; and 6 mg/mL on *A. sculptum*. All dilutions were prepared in a solution containing 1% acetone and 0.02% Triton X-100. The LIT was performed in duplicate (two technical replicates) at each concentration, including for the control group, and was repeated five times on different days (five biological replicates per treatment group). The control group was treated with a solvent solution of 1% acetone and 0.02% Triton X-100. Approximately 100 larvae were immersed in each concentration for 10 min. The tubes were immediately sealed, shaken for 5 s, and allowed to rest for 10 min. Afterwards, the larvae were placed on filter paper to dry and then transferred to a dry filter paper package (8.5 cm × 7.5 cm) and sealed with plastic clips. The packages were placed in a BOD incubator at a temperature of 27 ± 1 °C and relative humidity (RH) ≥ 80% for 24 h. After incubation, dead and live larvae were counted using a vacuum suction pump. Tick larvae that showed no movement after CO_2_ stimulation were considered dead.

### 4.4. Determination of Enzymatic Activity in Larvae

#### 4.4.1. Treatment and Processing of Larvae

Larvae of *A. sculptum* were treated with LC_5_, LC_25_, LC_50_, and LC_75_ of EO *P*. *aduncum* or Dillapiole. Approximately 1000 larvae were added to microcentrifuge tubes containing the lethal concentrations of EO *P. aduncum* or Dillapiole (shown in Table 2) for larval treatment. The tubes were immediately closed, shaken for 5 s, and then left to rest for 10 min. After this period, the larvae were dried on filter paper, transferred to filter paper packages (10 cm × 6 cm), and closed with clips. The packages were placed in a B.O.D. incubator at 27 °C and RH ≥ 80% for 24 h, and larvae that survived the treatments were collected for further processing.

Surviving larvae were immediately macerated in PBS buffer (NaCl 136.8 mM; KCl 2.7 mM; Na_2_HPO_4_ 4.76 mM; KH_2_PO_4_ 1.76 mM pH 7.4) with 0.1% Triton X-100 and centrifuged (12,000× *g*, 5 min at 4 °C). The supernatant was removed and transferred to another microcentrifuge tube, where a mixture containing protease inhibitors at final concentrations of 1 mM Pepstatin A, 2 mM PMSF, 1.5 mM EDTA, and 10 μM E64 was added. The tubes were then kept at −80 °C until the measurement of enzymatic activity. The total protein content of all extracts was quantified using the Bradford method [51].

A negative control was established using a diluent (1% acetone and 0.02% Triton X-100) to prepare samples from larvae not exposed to EO or Dillapiole. Blanks were prepared by replacing the 10 µL of sample with 10 µL of PBS with 0.1% Triton X-100. All test sample absorbances were subtracted from the average of the blank absorbance for enzyme activity assays. All enzyme activity assays below were performed in duplicate (two technical replicates) at each concentration, including for the control group, and were repeated five times (five biological replicates per treatment group) on different days.

#### 4.4.2. Glutathione-S-Transferase Activity Assay

The glutathione S-transferase (GST) activity of the larval protein extracts was determined according to the method described by Habig, 1974 [52]. The assay intended for the measurement of total GST activity is based upon the GST-catalyzed reaction between the substrates glutathione (GSH) and CDNB (1-chloro-2,4-dinitrobenzene).

Ten μL of protein extract from treated larvae was transferred to a plate (BIOFLOAT™ 96-Well Plate). Then, 190 μL of the mixture (100 μL of 100 mM potassium phosphate buffer, pH 6.5, 78 μL of distilled water, 2 μL of CDNB 50mM in methanol, and 10 μL GSH 50 mM) was added to the wells and the plate was kinetically read at 340 nm for 60 min at 30 s intervals at 30 °C using a Versamax ELISA plate reader (Molecular Devices, Versamax, Battle Creek, MI, USA). The initial rate of absorbance increase was registered and the product concentration was calculated using the molar extinction coefficient of 9.6 mM/cm for S-(2,4-dinitrophenyl glutathione). One unit (U) of enzymatic activity is defined as the amount of enzyme that produces 1 µmol of product per minute. The activity was expressed as µU/µg protein./

#### 4.4.3. Esterase Activity Assay

Esterase activity was evaluated with two different substrates: α-naphthyl acetate (for α-Esterase) and β-naphthyl acetate (for β-Esterase), according to the method described by van Asperen (1962) [53]. Ten μL of protein extract from treated larvae was transferred to a plate (Biofloat™ 96-Well Plate). Then, 190 μL mixture (100 μL of 40 mM sodium phosphate buffer, pH 7.2, 80 μL of distilled water, and 10 μL of 30 mM α -naphthyl or β -naphthyl acetate substrate in acetone, depending on the enzyme being studied, α or β-Esterase) was added.

To construct the standard curve, 10 μL of products in acetone (α- or β-naphythol) was added to each well in the following amounts: 0; 0.001; 0.0025; 0.005; 0.0075; 0.01; 0.015; 0.02 micromoles. Then, 190 μL of the mixture (100 μL of 40 mM sodium phosphate buffer, pH 7.2, 80 μL of distilled water, and 10 μL PBS 0.1% Triton X-100) was added to each well containing the products.

The plate was incubated for 30 min at 30 °C. After incubation, 50 μL of staining reagent (3.4% SDS and 0.3% Fast Blue) was added to each well of the plate and the plate was incubated for 5 min at 30 °C. The absorbance of the samples was measured at 570 nm using a Versamax ELISA plate reader (Molecular Devices, Versamax, Biloxi, MS, USA). The activity was expressed as µU/µg protein.

#### 4.4.4. Determination of Acetylcholinesterase Activity

Acetylcholinesterase activity (AChE) of the larval protein extracts was determined according to Li et al. (2005) [54], with some modifications. The reaction mixture consisted of 10 μL of 20 mM acetylthiocholine iodide, 100 μL of 100 mM sodium phosphate buffer, pH 7.8, and 80 μL of distilled water. A total of 190 μL of the mixture was transferred to a plate (BIOFLOAT™ 96-Well Plate), and 10 μL of protein extract from treated larvae was added to each well and incubated for 60 min at 30 °C. After incubation, 50 μL of staining reagent containing 2% SDS, 6 mM 5,5′-Dithiobis (2-nitrobenzoic acid) (DTNB) and 10 mM sodium phosphate buffer, pH 7,8, was added to each well and incubated for 5 min at 30 °C. The absorbance of the samples was measured at 410 nm using a Versamax ELISA plate reader (Molecular Devices, Versamax, Biloxi, MS, USA). The activity was expressed as Abs/min/ng protein.

### 4.5. Scanning Electron Microscopy (SEM)

Larvae of *A. sculptum* were sorted into the following treatment groups for a period of 24 h: Vehicle Control Group (1% acetone and 0.02% Triton X-100); LC_50_—*P. aduncum* Group; LC_75_—*P. aduncum* Group; LC_50—_Dillapiole Group; LC_75_—Dillapiole Group. Ten larvae from each group were subsequently fixed in a solution of 2% paraformaldehyde + 2% glutaraldehyde for 21 days. After fifteen days, the fixative was changed and left for an additional 5 days to complete the 21 days. Subsequently, the fixative solution was completely removed, followed by four washes in PBS buffer (15 min each), and placed in 70% alcohol for 24 h. The samples were dehydrated in a graded series of alcohol (80, 90, 95, and 100%) for 30 min each. In the final bath, the samples were transferred to adapted Eppendorf 1.5 mL tubes and placed inside a 15 mL Falcon tube with 100% alcohol, which was then sent to the Multi-User High-Resolution Microscopy Laboratory at the Federal University of Goias (LABMIC/UFG) for drying and image capture.

After dehydration, hexamethyldisilazane (HMDS) was applied for 5 min. Subsequently, excess HMDS were removed, and the Eppendorf tube was left open at room temperature until complete removal of the reagent. After drying, the samples were mounted on cylindrical “Stub” sample holders on double-sided copper conductive tape. Subsequently, the samples were coated with a conductive material, gold, using the Desk V Gold Film Deposition System, Denton Vacuum LLC, Moorestown, NJ, USA. The samples were then analyzed using a Scanning Electron Microscope (SEM) (JSM-6610, Jeol, Tokyo, Japan) equipped with EDS (Thermo Scientific NSS Spectral Imaging) at an acceleration voltage of 8 kV.

### 4.6. Statistical Analysis

The data were organized in spreadsheets using Microsoft Excel (Office 2007) software. To calculate the LCs, the data were initially transformed to *log*(X), normalized, and then the nonlinear regression was calculated to obtain LC_50_ (50% lethal concentration) using the GraphPad Prism 7.0 software (GraphPad Inc., San Diego, CA, USA). The LC_5_, LC_25_, and LC_75_ were estimated by using ECanything from LC_50_ (https://www.graphpad.com/quickcalcs/Ecanything1.cfm) access date 12 April 2023, Quick Calcs—GraphPad; EC (effective concentration); and entering the LC_50_ HillSlope of each compound (EO *P aduncum* or Dillapiole). Data distribution and normality were tested using the Kolmogorov–Smirnov test (*p* > 0.05). Comparisons between groups were carried out using a one-way ANOVA followed by Tukey’s Multiple Comparison post hoc test (*p* < 0.05).

## 5. Conclusions

The EO from *P. aduncum* and its major phenylpropanoid Dillapiole showed acaricidal activity in larvae of the tick *A. sculptum*. The potential increase in enzymatic activity (α-EST, β-EST, and GST) may serve as a response to minimize the damage caused by *P. aduncum* EO and Dillapiole. Conversely, examination of AChE after treatment with both *P. aduncum* EO and Dillapiole revealed a reduction in the activity of this enzyme at higher concentrations, suggesting the potential of these two products as substitute for synthetic pesticides such as organophosphates and carbamates. However, the *P. aduncum* EO and Dillapiole caused no damage to the tick cuticle. Additionally, *P. aduncum* EO and Dillapiole had a simultaneous effect on different detoxifying enzymes and on neurotransmission, which suggests that they trigger a complex response in *A. sculptum* and could potentially delay the development of resistance against these products. Further studies are needed to help clarify the exact mechanisms by which these compounds function and to strengthen the evidence supporting their potential to control ticks.

## Figures and Tables

**Table 1:** Chemical composition of the essential oil of *Piper aduncum*.

Compounds	RI^a^	RI^b^	%	Peak
α-Pinene ^S^	932	932	0.3	1
β-Pinene ^S^	975	974	0.3	2
β-Myrcene ^S^	992	988	0.1	3
α-Phellandrene ^S^	1004	1002	0.1	4
2-Carene ^S^	1010	1008	0.1	5
p-Cimene ^S^	1024	1020	0.1	6
β-Phellandrene	1028	1025	0.2	7
(*Z*)-β-Ocimene ^S^	1039	1032	1.6	8
(*E*)-β-Ocimene ^S^	1049	1044	3.4	9
γ-Terpinene	1059	1054	0.2	10
α-Terpinolene ^S^	1088	1086	0.4	11
Oxygenated monoterpene *	1209	-	0.1	12
(+)-Piperitone	1255	1249	0.7	13
δ-Elemene	1339	1335	0.1	14
α-Ylanglene	1374	1373	0.1	15
α-Copaene ^S^	1378	1374	0.2	16
β-Elemene	1394	1389	0.2	17
α-Gurjunene ^S^	1412	1409	0.1	18
(*E*)-β-Caryophyllene ^S^	1422	1417	0.8	19
β-Gurjunene	1432	1431	0.2	20
α-Humulene ^S^	1457	1452	0.9	21
Germacrene D	1484	1481	2.7	22
Bicyclogermacrene	1500	1500	2.3	23
α-Muurolene	1503	1500	0.1	24
α-Bulnesene	1510	1509	0.2	25
γ-Cadinene	1517	1513	0.1	26
Myristicin	1524	1518	1.2	27
δ-Cadinene	1522	1522	0.1	28
Germacrene B	1561	1559	0.2	29
(*E*)-Nerolidol ^S^	1566	1561	0.1	30
Spathulenol	1581	1577	0.1	31
Veridiflorol	1596	1592	0.3	32
Dillapiole ^S^	1632	1620	81.9	33
epi-α-Muurolol	1646	1640	0.1	34
α-Cadinol	1659	1652	0.2	35
Apiole ^S^	1686	1677	0.2	36

RI^a^—retention index calculated against C8-C40 n-alkanes using an HP-5ms column. RI^b^—retention index values from the literature (Adams 2007). ^S^ Compound identity confirmed with an authentic standard. The remaining compounds were identified by comparing the RI and mass spectra with the Adams and Wiley databases (see text for details). * Unidentified compound.

**Table 2:** Lethal concentrations (LC–mg/mL) of *Piper aduncum* and Dillapiole on *Amblyomma sculptum* larvae.

EO/Compound	LC_5_ *	LC_25_ *	LC_75_ *	LC_50_	HillSlope ± SE	CI 95%	R^2^
*Piper aduncum*	1.29	2.40	5.05	3.49	2.96 ± 0.14	3.36 to 3.62	0.95
Dillapiole	1.41	2.44	4.69	3.38	3.36 ± 0.21	3.24 to 3.54	0.93

* LC_5_, LC_25_, and LC_75_ were estimated using EC from EC_50_ (Quick Calcs—GraphPad) and the HillSlope value of LC_50_ for each population. LC_50_: lethal concentration (mg/mL) for 50% of individuals; SE: standard error; CI 95%: 95% confidence interval; R^2^: regression correlation coefficient.

## Data Availability

Data are available in the manuscript.
